# Knowledge, Perceptions, and Self-Reported Rates of Influenza Immunization among Canadians at High Risk from Influenza: A Cross-Sectional Survey

**DOI:** 10.3390/vaccines11081378

**Published:** 2023-08-17

**Authors:** Paul Roumeliotis, Sherilyn K. D. Houle, Ajit Johal, Bertrand Roy, Wendy Boivin

**Affiliations:** 1 School of Epidemiology and Public Health, University of Ottawa, Ottawa, ON K1N 6N5, Canada; 2 School of Pharmacy, University of Waterloo, Waterloo, ON N2L 3G1, Canada; sherilyn.houle@uwaterloo.ca; 3 Travelrx and Immunize.io, Vancouver, BC V5Z 3Y1, Canada; 4 CSL Seqirus Inc., Kirkland, QC H9H 4M7, Canada; bertrand.roy@seqirus.com

**Keywords:** influenza vaccination, vaccination coverage, adults 18–64 years, adults ≥ 65 years of age, chronic medical conditions

## Abstract

The Public Health Agency of Canada recommends that 80% of high-risk persons, including adults aged ≥65 years and 18–64 years with certain comorbidities, be vaccinated against influenza. During the 2022–2023 influenza season, we conducted an online survey of 3000 Canadian residents aged ≥18 years randomly recruited from the Léger Opinion (LEO) consumer panel to assess knowledge and perceptions about influenza vaccination as well as survey self-reported vaccination rates. Overall, 47.3% received an influenza vaccination during the 2022–2023 season. Vaccination rates among persons aged 18–64 years with high-risk medical conditions (n = 686) and among adults aged ≥65 years (n = 708) were 46.4% and 77.4%, respectively; 77.8% and 88.5%, respectively, believed influenza vaccination was important for people at high risk from influenza. Only 35.8% of adults aged 18–64 years with comorbidities were aware of being at high risk; 66.0% of this group was vaccinated against influenza, compared with 37.0% of those unaware of being at high-risk. During 2022–2023, 51.3% of people aged ≥65 years and 43.0% of people aged 18–64 years with comorbidities discussed influenza vaccination with healthcare providers. These findings suggest gaps in education regarding the importance of influenza vaccination among people at risk of influenza complications.

## 1. Introduction

Such as severe acute respiratory syndrome coronavirus 2 (SARS-CoV-2), the cause of coronavirus disease 2019 (COVID-19), the influenza virus often causes high rates of hospitalizations and deaths, especially in persons aged ≥65 years and those with chronic medical conditions that increase their risk of pneumonia and other cardiorespiratory complications [1,2,3]. In Canada, influenza and pneumonia are ranked among the 10 leading causes of death, and before the COVID-19 pandemic, influenza caused an average of 12,000 hospitalizations and 3500 deaths each year [4]. Rates of hospitalizations are highest among older adults, peaking at 280 per 100,000 during the high-severity 2017–2018 influenza season [5]. A study conducted in 2017 suggested that each influenza hospitalization is associated with $14,612 (Canadian dollars) in direct medical costs [6].

Given the burden of influenza, the Canadian National Advisory Committee on Immunization (NACI) recommends influenza vaccination for persons at high risk of influenza complications, including all pregnant individuals, residents of nursing homes and other chronic care facilities, and people with Indigenous Canadian ancestry. In addition, the NACI high-risk group includes adults aged ≥65 years and anyone with any of the following chronic health conditions: asthma or another chronic lung disease such as emphysema, chronic bronchitis, or cystic fibrosis; a heart condition such as coronary heart disease, heart failure, or myocardial infarction; hypertension; cancer; diabetes or other metabolic diseases; chronic liver disease; chronic kidney disease; immune disorder or immune suppression such as with chemotherapy, radiation, steroid use, or an organ transplant; spleen problems or removal; anemia, thalassemia, or hemoglobinopathy; morbid obesity (body mass index [BMI] > 40 kg/m^2^); conditions that compromise management of respiratory secretions, with increased risk of aspiration; or chronic cerebrospinal fluid (CSF) leak [4].

The US Advisory Committee on Immunization Practices (ACIP) also prioritizes influenza vaccination for individuals with a similar set of high-risk conditions. In addition, the ACIP recommends enhanced influenza vaccines—either adjuvanted or higher-dose influenza vaccines—for persons aged ≥65 years [7]. These vaccines were specifically designed to increase the immune response of older adults, in whom standard influenza vaccines may be less effective due to immunosenescence, frailty, and cumulative comorbidities [8]. Based on a literature review conducted in 2018, NACI does not specifically recommend enhanced influenza vaccines over standard vaccines for older adults [4,9]. Instead, NACI emphasizes the importance of influenza vaccination in general and leaves the choice of vaccine up to the healthcare provider [4]. However, NACI has communicated that an updated analysis is in progress [10].

To prevent influenza complications in at-risk groups, the Public Health Agency of Canada (PHAC) has set a vaccination coverage target of 80% by 2025 for adults aged ≥65 years and for those 18–64 years of age with NACI-defined high-risk medical conditions [11]. Even in years with low absolute vaccine effectiveness (usually due to a mismatch between circulating and vaccine viruses), vaccines reduce the rate of hospitalizations and deaths. For example, during the 2017–2018 season in the US, influenza vaccination was estimated to have prevented 65,000 hospitalizations among persons ≥ 65 years of age despite only 17% estimated effectiveness against influenza infection [12]. In addition, influenza vaccination has been shown to prevent cardiovascular events in people with cardiovascular disease [2,13].

Despite the benefits of vaccines, especially in high-risk populations, vaccination rates in Canada are well below PHAC-recommended targets [14]. The aim of the research reported here is to examine Canadians’ knowledge and perceptions regarding influenza immunization and assess self-reported rates of immunization, with particular focus on those considered to be at high-risk.

## 2. Materials and Methods

### 2.1. Study Design

This observational, cross-sectional, self-reported online survey consisted of a structured questionnaire that was answered online by 3000 Canadian residents aged ≥18 years between 5–21 December 2022. The questionnaire was available in English and French and was delivered electronically via multiple Internet-accessible platforms.

The study design was approved by the Veritas Independent Review Board (IRB). All survey participants provided informed consent that adhered to the IRB requirements and applicable laws and regulations. Respondent information was fully anonymized before data collection, aggregation, and analysis.

### 2.2. Participants

Eligible survey respondents resided in Canada and were ≥18 years of age. The study population was drawn from the Léger Opinion (LEO) consumer panel, which includes nearly 500,000 active members. Respondents were recruited in a random selection process that utilized traditional and mobile telephone methodologies to assemble a survey panel that was regionally and demographically representative of the Canadian population. Each prospective survey respondent accessed the survey through a unique survey link that was provided by Léger. Recruitment efforts continued during the study until the target of 3000 respondents was reached.

The LEO registration process is designed to prevent multiple entries and fraudulent panelists through the use of usernames and strict restrictions on passwords, a detailed profiling questionnaire for all panelists, and de-duplication routines to prevent existing panel members from joining more than once. Where consistent with local regulations, compensation equal to fair market value was offered to respondents who completed the survey.

A prespecified high-risk subgroup included respondents who met at least one of the following criteria: ≥65 years of age, pregnant, resident of a long-term care facility, Indigenous ancestry, or a high-risk medical condition as defined by NACI (i.e., hypertension, diabetes or other metabolic disease, asthma, other chronic lung disease, heart disease, body mass index [BMI] ≥ 40 kg/m^2^, anemia, immune disorder, cancer, or conditions that adversely affect respiratory secretions and/or increase the risk of aspiration).

### 2.3. Study Objectives

The objectives of the study were to understand Canadians’ attitudes and beliefs toward influenza vaccination in the 2022–2023 influenza season, gain insight into Canadians’ preferences and unmet needs around influenza vaccination, and explore the perceptions and uptake of influenza vaccination among individuals at high risk of influenza complications.

### 2.4. Survey Instrument

The online survey questionnaire consisted of approximately 70 questions (Supplementary Materials: Survey Instrument), including screening questions, introductory questions, or contextual updates. Survey domains included demographics, general attitudes toward vaccines, COVID-19 vaccination status, discussions with healthcare providers about influenza vaccines, influenza vaccine status, influenza vaccine experience at pharmacies, knowledge and awareness of enhanced influenza vaccines, and awareness of influenza vaccines specifically recommended for groups at high-risk of influenza or its complications. Most domains included multiple variables, and the number of questions seen by respondents varied depending on their answers, as some; however, not all, responses prompted follow-up questions.

The survey was hosted on the Decipher Survey Platform (Forsta, Vancouver, BC, Canada) and took approximately 15 min to complete. Assuming 90% of LEO panel members would meet study entry criteria and a response rate of 15%, ~22,000 panel members were invited to participate in the study to meet the target sample of 3000 respondents.

To ensure the representativeness of the sample, specific recruitment quotas by age, gender, and region were set based on 2021 Canadian census data [15]. Throughout the recruitment phase, sampling was adjusted to ensure the collected data were representative—for example, if a certain cohort was underrepresented, more respondents from that cohort were recruited. At the end of data collection, to further ensure representativeness, the data were weighted by age, region, and gender based on 2021 census data [15].

Respondents could access the survey on a website using a computer, smartphone, or tablet, or by using a proprietary app for mobile devices. To ensure respondents answered all questions before submitting the survey, the survey programming permitted advancement to the next question only after respondents clicked on an answer and then clicked on the ‘continue’ button on each screen. At the end of the survey, respondents clicked on a ‘finish’ button; those who answered all questions and clicked ‘finish’ were recorded as ‘complete’ in the system.

To avoid multiple entries, respondents who clicked on a survey were assigned a unique identifier that was linked to each respondent’s LEO panel account. The system recorded whether the respondent completed or was terminated (i.e., excluded) from the survey. If respondents attempted to take the survey again, the system recognized the unique ID and showed a message that they had already completed the survey.

### 2.5. Statistical Methodology

Assuming a 95% confidence level, an online survey of 3000 respondents was projected to yield a margin of error of ±1.79%, 19 times out of 20. Assuming 90% of LEO panel members would meet study entry criteria and a response rate of 15%, ~22,000 LEO panel members were invited to participate in the study to meet the target sample of 3000 respondents.

The analysis was performed using the statistical software IBM Q/SPSS (Version 29) and Microsoft Corporation, Excel (2023). Relationships within the data were primarily analyzed using cross-tabulation. Primary independent variables such as vaccination status and intent to become vaccinated were analyzed along with key demographic characteristics, including age, gender, and ethnicity. The statistical significance of cross-tabulations was tested using a z-test for proportions and a *t*-test for means, with a 95% confidence interval (CI). Because only descriptive statistics were reported, no formal sample size calculations were performed.

## 3. Results

### 3.1. Study Population

**Figure 1:**
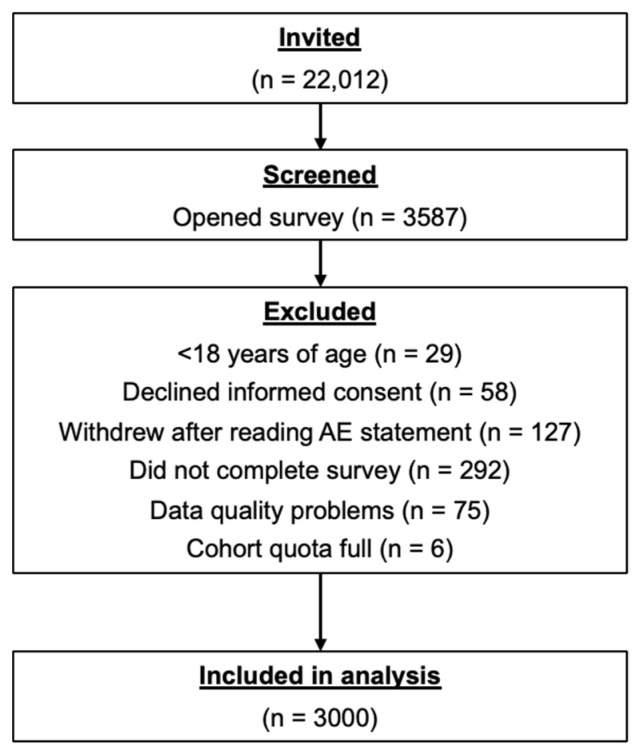
Disposition of survey participants. AE, adverse event.

Between 5–21 December 2022, 22,012 potential respondents were invited to participate; 3587 opened the survey, and the responses from 3000 survey participants were included in the analysis. Six respondents who completed the survey were excluded because their respective cohort had already reached its target number of participants. Other prospective survey participants did not meet entry criteria or complete the survey, and some were excluded due to data quality problems (Figure 1).

As shown in Table 1, in the weighted sample, 48.2% and 51.2% of respondents identified themselves as male and female, respectively (<1% identified as gender diverse). Nearly one-third of respondents were 35–54 years of age, and almost one quarter were ≥65 years of age. Most respondents were of White race (European ancestry); the next largest racial and ethnic group included people of Asian ancestry. Respondents were drawn from Canadian provinces and territories in proportions reflective of regional population sizes, and the proportions of the study sample were roughly similar across community sizes. Individuals with NACI-defined medical conditions accounted for 37.2% of the study population. The total high-risk cohort comprised 50.7% of the study population, including individuals who were ≥65 years of age, pregnant, of Indigenous ancestry, or long-term care residents, in addition to those with high-risk medical conditions.

### 3.2. Vaccination History

**Figure 2:**
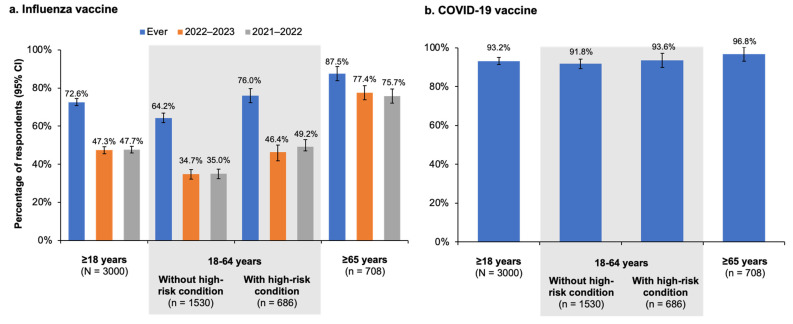
Proportion of respondents answering ‘Yes’ to questions about whether they had received an influenza vaccine at any time in the past (ever) or during the current (2022–2023) or previous (2021–2022) influenza seasons (**a**) or ever received at least one dose of a COVID-19 vaccine (**b**). Error bars represent the 95% confidence interval (CI).

The majority of respondents (72.6%) reported receiving an influenza vaccination at some point in the past, whereas 47.3% received one during the 2022–2023 influenza season and 47.7% during the 2021–2022 season (Figure 2a). Over 90% of all respondents, including those at and not at high risk from influenza or COVID-19, reported receiving at least one dose of a COVID-19 vaccine (Figure 2b).

**Figure 3:**
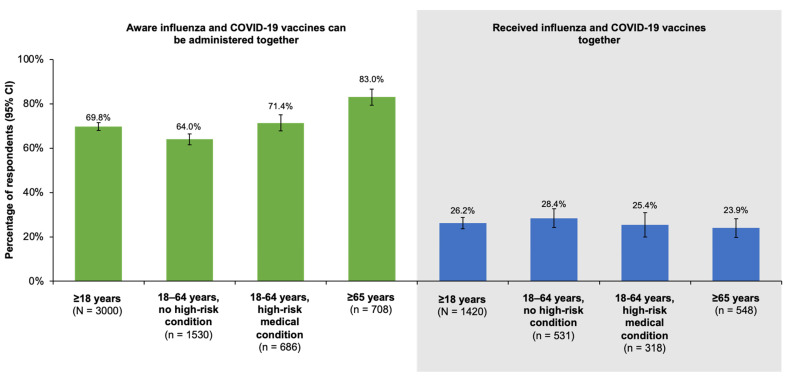
Proportion of respondents answering ‘Yes’ to the question, “Before today, were you aware that healthcare providers can administer the flu vaccine at the same time (or any time before or after) administering the COVID-19 vaccine?” (**left**) and the proportion of respondents who reported receiving an influenza vaccine during the 2022–2023 season who also received a COVID-19 vaccine or booster at the same time (**right**). Error bars represent the 95% confidence interval (CI).

As shown in Figure 3, although 69.8% (95% CI, 68.0–71.6%) of respondents were aware the influenza and COVID-19 vaccines could be co-administered, only 26.2% (95% CI, 23.6–28.8%) of respondents who reported receiving the influenza vaccine received a COVID-19 vaccine or booster at the same time. Compared with respondents who were not at risk, more respondents with a high-risk condition (either medical or age-related) were aware the vaccines could be co-administered; however, only slightly more high-risk respondents who reported receiving an influenza vaccine also received a COVID-19 vaccine at that time. Nevertheless, 53.1% (95% CI, 51.3–54.9%) of all respondents reported they would be interested in receiving the two vaccines together. Interest was higher among those aged 18–64 years with high-risk conditions (54.6% [95% CI, 50.9–58.3%]) and highest among respondents aged ≥65 years (69.9% [95% CI, 66.2–73.6%]).

### 3.3. Respondents at High Risk from Influenza

Out of the entire study population, 1593 (53.1% [95% CI, 51.3–54.9%]) respondents were aware that NACI specifically recommends the influenza vaccine for persons at high risk of influenza complications (see Table 1). Of the 1515 individuals included in the overall high-risk population, 80.6% (95% CI, 78.1–83.1%) reported being vaccinated against influenza at some point in the past, whereas 59.5% (95% CI, 57.0–62.0%)) reported being vaccinated during the 2022–2023 season. Out of the total high-risk cohort, 43.2% (95% CI, 40.7–45.7%) were aware they belonged to a group for whom NACI specifically recommended influenza vaccination. Because only 9.2% of respondents were pregnant, resided in a long-term care facility, or had Indigenous ancestry, further analysis was focused on the subgroups who were 18–64 with a high-risk medical condition or those aged ≥65 years.

#### 3.3.1. Respondents Aged 18–64 Years with a High-Risk Medical Condition

**Figure 4:**
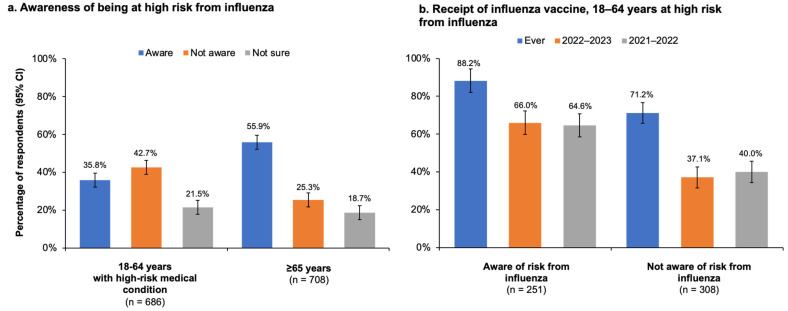
(**a**) Responses to the question, “As far as you know, do you belong to a high-risk group for whom influenza vaccine is particularly recommended?” by persons aged 18–64 years with a high-risk medical condition and by persons aged ≥65 years. (**b**) Proportions of at-risk respondents aged 18–64 years who reported receiving the influenza vaccine at any time in the past (ever) or during the current (2022–2023) or previous (2021–2022) influenza seasons. Error bars represent the 95% confidence interval (CI).

Of the 686 persons aged 18–64 years with a high-risk medical condition, 395 (57.5% [95% CI, 53.8–61.2%]) were aware of the NACI recommendation that persons at high risk should receive the vaccine, and 72.5% (95% CI, 68.8–76.2%) of this group stated that they were somewhat (53.4% [95% CI, 49.7–57.1%]) or very knowledgeable (19.1% [95% CI, 15.4–22.8%]) about the recommendation. In addition, 77.8% (95% CI, 74.1–81.5%) of all high-risk adults younger than 65 years reported that it was important for people with a high-risk condition to receive the influenza vaccine. However, most respondents aged 18–64 who met NACI criteria were not aware they fell into the high-risk category (Figure 4a). Those who were aware of their high-risk status were more likely to have been vaccinated against influenza than those who were not aware (Figure 4b).

Less than half of respondents aged 18–64 years with a high-risk medical condition (43.0% [95% CI, 40.5–45.5%]) had spoken with a healthcare provider about the influenza vaccine during the 2022–2023 season, although even lower proportions had discussed influenza vaccination with a healthcare provider in the overall study population (39.1% [95% CI, 37.3–40.9%]) or among those aged 18–64 years without a high-risk condition (32.3% [95% CI, 31.0–33.6%]).

Small minorities of medically at-risk persons aged 18–64 years who reported they did not receive the influenza vaccine (n = 204) did so because they were “healthy and/or never get the flu” (5.8%) or were “healthy, not at risk of influenza-related complications or hospitalization” (3.4%). The most common reasons among this group for not being vaccinated were “no specific reason” (19.9%) or the respondent “did not get around to it” (16.9%). “Concerns about the influenza vaccine and/or its side effects” were reported by 13.6% of high-risk persons aged 18–64 years who were unvaccinated, and 3.4% and 2.2% of this cohort reported “flu vaccines don’t work” and “I don’t believe in vaccines,” respectively.

#### 3.3.2. Respondents Aged ≥65 Years

As shown in Figure 2a, more than three-quarters of the 708 respondents aged ≥65 or older reported receiving the influenza vaccine during the previous two seasons. Awareness of NACI recommendations for influenza vaccination for high-risk persons was similar among adults aged 65 years and older (59.8% [95% CI, 56.1–63.5%]) and those younger than 65 years with high-risk conditions (57.5% [95% CI, 53.8–61.2%]); however, a higher percentage of older adults (88.5% [95% CI, 84.8–92.2%] vs. 77.8% [95% CI, 74.1–81.5%]) believed it was important for people at high risk to be vaccinated against influenza. Nevertheless, only 55.9% (95% CI, 53.8–61.2%) of older respondents reported that they themselves were at high risk from influenza. Just over half (51.3% [95% CI, 47.6–55.0%]) of older adults had spoken to a healthcare provider about the influenza vaccine during the 2022–2023 influenza season; however, 63.5% (95% CI, 59.8–67.2%) had never had a discussion with their healthcare provider about enhanced vaccines specifically designed for adults aged ≥65 years.

Despite this gap in communication, when asked whether they were aware of the existence of different vaccines for older vs. younger adults, 67.3% (95% CI, 63.6–71.0%) of the older age group answered ‘Yes,’ and 51.3% (95% CI, 47.6–55.0%)) reported knowing there was more than one type of enhanced vaccine made specifically for adults ≥ 65 years of age. In addition, 69.3% (95% CI, 65.6–73.0%) of this group agreed with the statement, “Enhanced vaccines help better protect older adults from the seasonal flu.”

**Figure 5:**
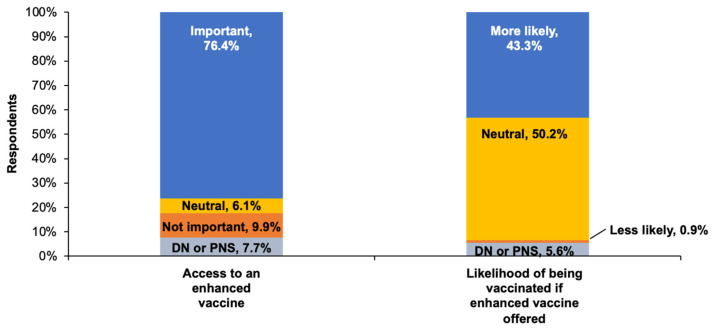
Responses of persons aged ≥65 years (n = 708) to the following questions: “How important is it for you to have access to an enhanced influenza vaccine?” (**left column**) and “If your province/territory offered enhanced influenza vaccines, would it make you more likely to get vaccinated against the flu?” (**right column**). DN = don’t know; PNS = prefer not to say.

As shown in Figure 5, 76.4% (95% CI, 67.1–74.5%) of adults ≥ 65 years reported that personal access to enhanced vaccines was important to them, while 43.3% (95% CI, 39.6–47.0%) reported they would be more likely to be vaccinated against influenza with an enhanced vaccine if it were offered.

Only 71 (22.6%) respondents aged ≥65 years reported not receiving any influenza vaccine during the 2022–2023 season. The pattern and frequency of reasons given by older respondents for not being vaccinated were similar to those given by younger respondents with high-risk medical conditions. “No specific reason” was cited by 18.9% of the ≥65 group, and “did not get around to it” by 12.5%, although 16.2% were concerned about the vaccine or its side effects. Few unvaccinated older persons cited “flu vaccines don’t work” (2.8%) and “I don’t believe in vaccines” (1.4%) as their reasons for not being vaccinated.

## 4. Discussion

In this survey study conducted during the 2022–2023 influenza season, 72.6% (95% CI, 70.8–74.4%) of the 3000 Canadian residents aged ≥18 years who participated reported receiving an influenza vaccine at some point in the past, and >90% reported receiving at least one dose of the COVID-19 vaccine. However, less than half of respondents reported receiving the influenza vaccine during the current (2022–2023; 47.3% [95% CI, 45.5–49.1%]) or previous (2021–2022; 47.7% [95% CI, 45.9–49.5%]) influenza seasons. Influenza vaccination rates among respondents at high risk for influenza were higher, with 80.6% (95% CI, 78.1–83.1%) reporting being vaccinated at some point and 59.5% (95% CI, 57.0–62.0%) and 60.1% (95% CI, 57.6–62.6%) reporting receiving the vaccine during the current and previous seasons, respectively. Among the high-risk population, adults ≥65 years of age were the most likely to state they were vaccinated against influenza in the past (87.5% [95% CI, 83.8–91.2%]) or during the current and previous seasons (77.4% [95% CI, 73.7–81.1%] and 75.7% [95% CI, 72.0–79.4%], respectively). Younger adults with high-risk medical conditions were vaccinated at lower rates, with 76.0% (95% CI, 72.3–79.7%) reporting ever being vaccinated and 46.4% (95% CI, 42.7–50.1%) and 49.2% (95% CI, 45.5–52.9%) vaccinated in the 2022–2023 and 2021–2022 seasons, respectively. Younger adults not at high risk had the lowest reported vaccination rate, with only 34.7% (95% CI, 32.2–37.2%) and 35.0% (95% CI, 32.5–37.5%) stating they were vaccinated in the past two seasons (≤35.0% in the past two seasons and 64.2% (95% CI, 61.8–66.8%) in any season.

The vaccination rates reported in our study for the 2021–2022 season were higher than those reported in the most recent PHAC survey, which found that 39% of all adults, 27% of adults aged 18–64 years without high-risk conditions, 38% of the 18–64 year age group with high-risk conditions, and 71% of adults ≥65 years of age were vaccinated against influenza [14]. Both our results and PHAC survey findings for adults aged 18–64 years with high-risk conditions are considerably lower than—indeed, almost half of—the PHAC 80% vaccination coverage target by 2025 [11]. In our study, while the majority of high-risk adults younger than 65 years knew that NACI recommends influenza vaccination for certain risk groups [4], only 35.8% (95% CI, 32.1–39.5%) were aware of their own high-risk status. It is possible this lack of awareness may have contributed to the low rate of vaccination in younger adults. Nevertheless, the high-risk population younger than 65 years was still more likely to be vaccinated than those in the same age group who were not at risk. Moreover, 77.8% (95% CI, 75.3–80.3%) of adults with a high-risk condition agreed that influenza vaccination was important for those at-risk, and of those not vaccinated during the current season, only 13.6% expressed concern about influenza vaccines and 3.4% expressed skepticism about influenza vaccination effectiveness.

The vaccination rates reported in this study may be considered an indicator of respondents’ knowledge and perceptions about vaccination. Vaccine hesitancy due to concerns about vaccine safety or disagreement with the concept of vaccination was uncommon among respondents in our survey, a finding that is consistent with other recent surveys among Canadians [14,16,17]. However, the finding that less than half of respondents had received an influenza vaccine during the current season, while most had received one at some point in their lives, suggests that many Canadians lack awareness about the benefits of influenza vaccines. The plurality of unvaccinated respondents in our survey had no specific reason for not receiving the vaccine or reported they had not made time to conduct it. These findings suggest that education regarding the importance of vaccination, coupled with convenient and accessible access to the vaccine, may help improve vaccine coverage rates, especially if these efforts are directed at high-risk individuals. Our study results also point to a communication gap between healthcare providers and their patients, as only 43% (95% CI, 39.3–46.7%) of high-risk adults aged 18–64 years and 51.3% (95% CI, 47.6–55.0%) of adults aged ≥65 years had spoken with their healthcare provider about influenza vaccines during the previous season. Another study evaluating trends in influenza vaccination among Canadians with cardiovascular disease based on the Canadian Community Health Survey (CCHS) between 2009 and 2018 found similar gaps in communication as well as suboptimal rates of vaccination in a high-risk population. Even though participants in this study who had a healthcare provider were more than twice as likely to be vaccinated against influenza as those who didn’t have a healthcare provider, vaccination rates hovered close to 60% over the five studied seasons [17]. The Canadian Influenza Immunization Awareness Campaign provides a variety of educational resources on influenza that target at-risk groups such as older adults, pregnant women, Indigenous people, individuals with asthma, and those with unspecified chronic diseases [18]. Perhaps awareness campaigns explicitly focused on NACI-defined high-risk conditions, along with efforts to encourage healthcare providers to emphasize the importance of influenza vaccines for patients with these conditions, will help raise vaccination rates in this population.

Vaccination rates among older adults aged ≥65 years participating in our survey came much closer to the PHAC 80% vaccination coverage target, with the overall (i.e., at any point in the past) rates surpassing the target and the current and previous season rates nearly reaching the target. Compared with adults younger than 65 years (with or without high-risk conditions), older adults were not only more likely to report being vaccinated but also to believe in the importance of high-risk groups receiving an influenza vaccine. These findings are supported by the CCHS study of vaccination coverage among people with cardiovascular disease, which showed that adults aged ≥65 years were four times more likely than younger adults to be vaccinated against influenza, even though all age groups studied were at high risk of influenza [17]. Another study based on data from the Canadian Longitudinal Study on Aging (CLSA) also found that older adults were 3–4 times more likely to be vaccinated against influenza compared with younger adults [16]. Notably, even though vaccination rates were relatively high among older adults in our study, 44% did not know or were not sure that they themselves were at risk of influenza complications and thus belonged to a group for which influenza vaccination was specifically recommended—another finding pointing to a communication gap between healthcare providers and their patients and one that is also consistent with the CCHS study of influenza vaccine coverage among Canadians with cardiovascular disease [17].

Our survey did not inquire about the types of vaccines respondents had received during the 2022–2023 season, so it is unknown whether older vaccine recipients who participated in the survey were given a standard influenza vaccine or an enhanced one specifically designed for their age group. Most older respondents (67.3% [95% CI, 63.6–71.0%]) were aware that enhanced vaccines designed for adults ≥65 years old existed. Among those who were aware of enhanced vaccines, 53% said they had discussed them with a healthcare provider. Together, our findings in both the younger and older high-risk groups suggest a need for more education and communication between public health officials, healthcare providers, and the public about the importance of influenza vaccination for people at risk of complications.

With regard to COVID-19 vaccination, reported rates of coverage with at least one dose were >90% in our survey. Less than 30% of all groups studied, however, stated they had received their influenza and COVID-19 vaccinations together in the 2022–2023 season. The low rates of co-administration were not associated with a lack of awareness, as 69.8% (95% CI, 68.0–71.6%) and 83.0% (95% CI, 79.3–86.7%) of older adults were aware the two vaccines could be given at the same time. The majority of respondents also expressed interest in co-administration, especially the ≥65 year old age group, of which 69.9% (95% CI, 66.2–73.6%) reported interest in receiving the vaccines together. In a recent study of vaccine acceptance among Canadians before and during the COVID-19 pandemic based on CLSA data, the authors found that prior history of influenza vaccination was the strongest predictor of acceptance of both influenza and COVID-19 vaccines [16].

Because this study was designed as a survey, it is limited by the collection of information based on the perceptions of the participating respondents, and as such, there is no source data verification. A further limitation of this study is that, being cross-sectional, it cannot be used to demonstrate cause-and-effect relationships. The quality of data also depends, to a large extent, on the accurate reporting of information by respondents, which may be subject to recall bias.

## 5. Conclusions

We found high rates of influenza and COVID-19 vaccination interest and general acceptance among Canadians surveyed during the 2022–2023 influenza season; however, reported vaccination coverage rates fell short of PHAC targets, especially among individuals aged 18–64 years with medical conditions putting them at high risk of influenza complications. Those not vaccinated expressed concern regarding influenza vaccines and their effectiveness. Older adults aged ≥65 years were more likely to state they were vaccinated against influenza; however, this group also did not meet the PHAC target of 80% coverage. Although most survey respondents were aware NACI specifically recommends influenza vaccination for persons at high risk from influenza complications, the majority of respondents who were younger than 65 years with high-risk medical conditions, and close to half of those aged ≥65 years, did not view themselves as meeting high-risk criteria. These findings suggest a need for further interaction and education with healthcare providers and targeted public health outreach to at-risk populations regarding the benefits of influenza immunization.

## Figures and Tables

**Table 1:** Demographics of the study population (N = 3000).

Category	Characteristic	n (%)	
		Weighted	Unweighted
Gender	Male	1447 (48.2)	1422 (47.4)
	Female	1537 (51.2)	1565 (52.2)
	Other, nonbinary, or not specified	16 (0.6)	13 (0.4)
Age	18–24 years	302 (10.1)	267 (8.9)
	25–34 years	498 (16.6)	464 (15.5)
	35–44 years	495 (16.5)	497 (16.6)
	45–54 years	471 (15.7)	551 (18.4)
	55–64 years	526 (17.5)	529 (17.6)
	65–74 years	409 (13.6)	490 (16.3)
	≥75 years	299 (10.0)	202 (6.7)
Race and ethnicity	White	2328 (77.6)	2356 (78.5)
	Black	82 (2.7)	77 (2.6)
	Asian	422 (14.1)	390 (13.0)
	Indigenous	94 (3.1)	92 (3.1)
	Latin American	25 (0.8)	25 (0.8)
	Other	90 (3.0)	81 (2.7)
	Prefer not to say	52 (1.7)	50 (1.7)
Region	Alberta	334 (11.1)	329 (11.0)
	Atlantic region (New Brunswick, Newfoundland, Nova Scotia, Prince Edward Island)	202 (6.7)	237 (7.9)
	British Columbia	416 (13.9)	392 (13.1)
	Manitoba	107 (3.6)	118 (3.9)
	Ontario	1162 (38.7)	1150 (38.3)
	Quebec	692 (23.1)	675 (22.5)
	Saskatchewan	85 (2.8)	96 (3.2)
	Northwest Territories, Nunavut, Yukon	2 (0.06)	3 (0.1)
Community size	Rural (population < 50,000)	717 (23.9)	747 (24.9)
	Small town (population 50,000–250,000)	770 (25.7)	756 (25.2)
	Large city (population 250,000–1,000,000)	836 (27.9)	834 (27.8)
	Metropolis (population ≥ 1,000,000)	638 (21.3)	625 (20.8)
	Don’t know/not sure	29 (1.0)	27 (0.9)
	Prefer not to say	11 (0.4)	11 (0.4)
High-risk medical condition	Any	1116 (37.2)	1130 (37.7)
	Anemia, thalassemia, hemoglobinopathy	74 (2.5)	75 (2.5)
	Asthma	245 (8.2)	244 (8.1)
	BMI > 40 kg/m^2^	102 (3.4)	109 (3.6)
	Cancer	68 (2.3)	65 (2.2)
	Chronic CSF leak	3 (0.1)	3 (0.1)
	Chronic lung disease ^2^	59 (2.0)	58 (1.9)
	Chronic kidney disease	34 (1.1)	33 (1.1)
	Chronic liver disease	17 (0.6)	17 (0.6)
	Diabetes or other metabolic diseases	275 (9.2)	276 (9.2)
	Heart disease ^1^	121 (4.0)	117 (3.9)
	Hypertension	599 (20.0)	605 (20.2)
	Immune disorder or immune suppression ^4^	71 (2.4)	70 (2.3)
	Respiratory secretion impairment ^3^	36 (1.2)	33 (1.1)
	Spleen problems or removal	3 (0.1)	3 (0.1)
Other high-risk group	≥65 years	708 (23.6)	692 (23.1)
	Pregnant	72 (2.4)	70 (2.3)
	Long-term care resident	108 (3.6)	104 (3.5)
	Indigenous ancestry	94 (3.1)	92 (3.1)

^1^ Including coronary heart disease, heart failure, and heart attack. ^2^ Including emphysema, chronic bronchitis, or cystic fibrosis. ^3^ Including increased risk of aspiration. ^4^ Including chemotherapy, radiation, steroid use, or an organ transplant. Abbreviations: BMI, body mass index; CSF, cerebrospinal fluid.

## Data Availability

The authors confirm that the data supporting the findings of this study are available within the article and Supplementary Materials.

## Supplementary Materials

The following supporting information can be downloaded at: https://www.mdpi.com/article/10.3390/vaccines11081378/s1: Survey Instrument.
