# Supplementary material for: Knowledge, Perceptions, and Self-Reported Rates of Influenza Immunization among Canadians at High Risk from Influenza: A Cross-Sectional Survey
Source: Vaccines (Basel). 2023 Aug 17;11(8):1378. doi: 10.3390/vaccines11081378 (PMC10459598; doi:10.3390/vaccines11081378)
Supplement: Supplementary file 1 [file vaccines-11-01378-s001.zip › vaccines-2482189-supplementary.pdf]

Supplement to:

Knowledge, Perceptions, and Self-Reported Rates of Influenza Immunization among Canadians at High Risk from Influenza: A Cross-Sectional Survey

Final Survey Questionnaire on Influenza Vaccination

Thank you for agreeing to participate in our survey. Please rest assured that your answers will remain confidential. The survey will take approximately 12 minutes to complete.

SHOW ON A SEPARATE PAGE

Title of the study: Understanding Influenza Vaccination Uptake During the COVID-19 Pandemic in Canada

INTRODUCTION

This consent form is only part of the process of informed consent. It should give you the basic idea of what the research is about and what your participation will involve. If you would like more detail about something mentioned here, or information not included here, you should feel free to ask. Please take as long as you need to read this form carefully and to understand any accompanying information. If you consent, you will be able to print this information at the end for your record.

PURPOSE OF THE STUDY

The purpose of the survey research is to conduct an on-line survey of Canadians to better understand the persistent influenza vaccination gap.

PARTICIPATION AND TERMINATION

This research survey is being conducted by Leger, an independent market research agency working within specific Market Research Codes of Conduct, on behalf of a pharmaceutical company. This research survey should take about **12 minutes** to complete.

Your participation in this study is voluntary.

You may refuse to participate. If you choose to participate, you can change your mind at any time and withdraw from the study. Refusal to participate or withdrawal from the study will not compromise your

ongoing medical care. If you wish to end your participation, you can simply stop answering the questions.

RISKS

During your participation in this research survey, Leger (the agency conducting the survey) will collect information about you which will include your personal information and, if applicable, may include health related information. Personal information collected will be used only for purposes of this research survey. As data is collected online, there is always a small risk of a confidentiality breach; however, Leger takes extensive measures to ensure data protection and data security in conformity with the highest industry standards and has put in place very strict protocols involved in dealing with confidential information. Leger has a full operational infrastructure in-house which means they do not need to outsource or offshore any work. Leger's research data does not leave Canada at any time.

BENEFITS

The information collected will help to better understand the gap in influenza vaccination uptake during the COVID-19 pandemic in Canada.

COMPENSATION

You will receive 1,200 LEO points from Leger Marketing when you submit the survey.

CONFIDENTIALITY

Personal information collected in this research survey will be used only for purposes of this research. The results of this research will be reported in de-identified form only and will not identify you individually. Any information that identifies you with respect to this research study will be kept confidential. This research is subject to applicable laws governing the protection of Personal Information and your information will be treated confidentially. If you wish to withdraw after you have completed the survey, you shall notify Leger in order to remove the data you provided. The data will be removed and destroyed.

If you disclose information having to do with the safety or quality of Sponsor's products, the agency may be required to disclose non-identifiable information about you (e.g., gender) to the Sponsor as necessary to fulfil regulatory and legal obligations.

In the event the results from this research will be published, no information that may identify you individually will be included in any publication or presentation.

CONTACT

For any additional information regarding the research, please contact the Research Project Manager Bertrand Roy from Seqirus at:
Email: Bertrand.roy@seqirus.com

For any questions/issues regarding the survey, you may contact Dima Ostrikov from Leger Marketing at:
Email: dostrikov@leger360.com

This study has been reviewed by Veritas IRB. If you have any questions about your rights as a research participant or the Investigator's responsibilities, you may contact Veritas IRB 24 hours per day and 7 days per week at 514-337-0442 or toll-free at 1-866-384-4221. An Independent Review Board (IRB) is a group of scientific and non-scientific individuals who perform the initial and ongoing ethical review of the research study with the participant's rights and welfare in mind. If you have any study-related comments, complaints or concerns, you should first contact the study investigator. Please call the IRB if you need to speak to a person independent from the Investigator and the research staff, and/or if the Investigator and the research staff could not be reached.

CONSENT

By selecting "I Consent" below, you certify that you are eighteen (18) years old or older. You acknowledge and confirm you understand that all survey materials are proprietary to the survey owner and must not be used for any other purpose than participation in the survey or disclosed to any third party without the express written permission of the survey owner.

□ I consent.
☐ I do not consent. [Terminate]

You can print this form for your records if you wish by pressing the "Print This Form" button below before proceeding.
□ Print this form.

SCREENER

S1. Please provide your age.

_____ years
Prefer not to say **[TERMINATE]**

S2. In which province or territory do you live?

British Columbia	1
Alberta	2
Saskatchewan	3
Manitoba	4
Ontario	5
Quebec	6
New Brunswick	7
Nova Scotia	8
Prince Edward Island	9
Newfoundland	10
Nunavut	11
Northwest Territories	12
Yukon	13

S3. What is your gender?

Male	1
Female	2
Non-binary	3
Other (specify): ______________	96
Prefer not to say	99

Adverse Event Statement

This research has been commissioned by a company that manufactures medicines/medical devices. It is a legal requirement that the company keep records of any Adverse events (AE) or Product technical complaints (PTC) that people may have about their medicines/medical devices. We must assist the company in meeting its legal and regulatory obligations. Therefore, if, during the interview, you make any reference to an AE or PTC in relation to a medicine/medical device, we will let the company know about them even if it has already been reported by you directly to the company or the regulatory authorities.

In such a situation you will be asked whether or not you are willing to waive the confidentiality given to you under Market Research Codes of conduct specifically in relation to that AE or PTC. You can decide whether or not to give the company your name and contact details. If you do provide your name and details with the AE/PTC, please rest assured everything else you say during the course of the interview will continue to remain confidential, and you will still have the option to remain anonymous if you so wish.

Please indicate your response by selecting the appropriate option below.

I would like to proceed and give permission for my contact details to be passed on to the Drug Safety department of the company if an adverse event / product complaint is mentioned by me during the survey.	1	**CONTINUE**
I would like to proceed but do not wish for my contact details to be passed on to the Drug Safety department of the company if an adverse event / product complaint is mentioned by me during the survey.	2	**CONTINUE**
I don’t want to proceed and wish to end the survey here.	3	**TERMINATE**

MAIN QUESTIONNAIRE

SECTION A: VACCINATION IN GENERAL

A1. As far as you know, are you up to date on your recommended vaccines?

Yes	1
No	2
Don't know/Not sure	98
Prefer not to say	99

A2. Please indicate the extent to which do you agree or disagree with the following statements about vaccines in general.

		Strongly disagree	Somewhat disagree	Somewhat agree	Strongly agree	Don't know/Not sure	Prefer not to say
1	In general, I consider vaccines to be important for my health	1	2	3	4	98	99
2	I know enough about vaccines to make an informed decision about getting vaccinated	1	2	3	4	98	99

SECTION B: COVID-19 Vaccination

The next few questions will be around COVID-19 and your COVID-19 vaccination status…

B1. Have you been vaccinated against COVID-19?

Yes, one dose	1
Yes, two doses	2
Yes, three doses or more	3
No, but intend to	4
No, and do not intend to	5
Prefer not to say	99

B2. How likely are you to get an additional or booster dose of a COVID-19 vaccine if you are eligible to receive one?

Very unlikely	1
Somewhat unlikely	2
Somewhat likely	3
Very likely	4

I already booked an appointment for my booster dose	5
Don't know/Not sure	98
Prefer not to say	99

PN: ASK IF CODES 1, 2 OR 98 SELECTED AT B2

B3. Please tell us why you are **[PN: INSERT B2 RESPONSE]** whether you will get an additional or booster dose of a COVID-19 vaccine? (Open-ended)

B4. Canadian public health authorities have said getting a flu vaccine at the same time as a COVID-19 booster is safe and effective for adults. If you could receive a COVID-19 booster shot and a flu vaccine at the same time, would you?

Yes, definitely	1
Yes, probably	2
Probably not	3
Definitely not	4
Don't Know/Not sure	98
Prefer not to say	99

PN: ASK IF CODE 1 OR 2 SELECTED AT B4

B5. Why did you say that you **[INSERT "definitely" IF CODE 1 & "probably" IF CODE 2]** would receive a COVID-19 booster shot and a flu vaccine at the same time?

PN: ASK IF CODE 3 OR 4 SELECTED AT B4

B6. Why did you say that you would **[INSERT "probably" IF CODE 3 & "definitely" IF CODE 4]** not receive a COVID-19 booster shot and a flu vaccine at the same time?

B7. If public health authorities recommend that Canadians receive another COVID-19 booster shot this fall, what would be your vaccination intention?

I will get both a COVID-19 booster and a flu shot in one visit	1
I will get both a COVID-19 booster and a flu shot, but at different times	2
I will get a COVID-19 booster but not a flu shot	3

I will get a flu shot but not a COVID-19 booster	4
I will get neither a COVID-19 booster nor flu shot	5
Unsure	98

B8. How, if at all, has your overall view of vaccines changed since the COVID-19 pandemic began?

Overall, my view of vaccines has become …

Much more positive	1
A little more positive	2
Has not changed	3
A little more negative	4
A lot more negative	5
Don’t Know/Not sure	98
Prefer not to say	99

SECTION C: ADULT — Flu vaccination

We would now like to ask you some questions about the flu vaccine, and your vaccination status.

C1. Have you ever received the flu vaccine?

Yes	1
No	2

PN: ASK IF CODE 1 SELECTED AT C1

C2. Did you receive the flu vaccine last fall (that is, between September and December of 2021)?

Yes	1
No	2

PN: ASK IF CODE 1 SELECTED AT C1

C3. Have you ever received the flu vaccine in previous years (that is, in the fall of 2020 or before)?

Yes	1
No	2

PN: ASK IF CODE 1 SELECTED AT C3

C4. How many times?

Once	1
2-5 times	2
More than 5 times	3

PN: ASK IF CODE 1 SELECTED AT C2 OR C3

C5. Where did you receive your last flu shot? (Select only one)

Place of employment	1
Pharmacy	2
Physician's office or medical clinic	3
Hospital	4
Community-based public health clinic, such as in a shopping mall or library / CLSC **[ONLY FOR QC]**	5
Public Health Department	6
Other (Specify)	96
Can't remember	98
Prefer not to say	99

PN: ASK IF CODE 1 SELECTED AT C1

C6. Where would you prefer to get a flu shot? (Select as many as 3)

Place of employment	1
Pharmacy	2
Physician's office or medical clinic	3
Hospital	4
Community-based public health clinic, such as in a shopping mall or library / CLSC **[ONLY FOR QC]**	5
Public Health Department	6
Other (Specify)	96
Can't remember	98
Prefer not to say	99

PN: ASK IF CODE 1 SELECTED AT C2

C7. What are the reasons you decided to receive the flu vaccine last fall (that is, between September and December of 2021)? (Select all that apply)

PN: RANDOMIZE

I am worried about getting both the flu and COVID-19	1
I want to prevent infection / I do not want to get sick	2
I am at risk because of my health condition	3
I am at risk because of my age	4
I receive it every year / It's just something I've always done	5
To protect the health of others	6
It's recommended by my health care provider	7
It was encouraged by family members, colleagues or friends	8
It's free	9

Other (specify): ______________________	96
Don’t know/Not sure	98
Prefer not to say	99

PN: ASK IF CODE 2 SELECTED AT C2

C8. What was the *most important* reason why you did not receive the flu vaccine last fall (that is, between September and December of 2021)?

PN: RANDOMIZE

I don’t believe in vaccines	1
Flu vaccines don’t work	2
I am healthy, and/or never get the flu	3
Getting the flu doesn’t make me that sick	4
I did not get around to it	5
I have concerns about the flu vaccine, and/or its side effects	6
I have concerns about being exposed to COVID-19 while getting the flu vaccine	7
I got the flu before I had the opportunity to get the flu shot	8
It was too expensive (cost of the vaccine)	9
I was not able to get an appointment	10
No specific reason, I just didn’t get it	11
Other (specify): ____	96
Don’t know/Not sure	98
Prefer not to say	99

C9. Has the COVID-19 pandemic made it more or less likely that you will get the flu shot this coming fall (that is, between September and December of 2022)?

Much less likely	1
Somewhat less likely	2
No change in likelihood	3
Somewhat more likely	4
Much more likely	5
Don’t know/Not sure	98
Prefer not to say	99

PN: ASK IF CODE 4 OR 5 SELECTED AT C9

C10. Please tell us why the COVID-19 pandemic has made it more likely that you will get the flu shot this coming fall (that is, between September and December of 2022)? (Select all that apply)

PN: RANDOMIZE

I’ve seen how sick people can get from a virus	1
I want maximum protection against all viruses	2
I don’t want to get flu symptoms and have to get a COVID test	3
I don’t want to get flu symptoms and have to quarantine	4

I don’t want to get sick with both COVID-19 and the flu	5
I don’t want to have to miss work or school	6
To keep me as healthy as possible in case I come into contact with COVID-19	7
Getting the flu shot could help make my COVID-19 vaccine more effective	8
Other (specify)	9
Don’t know/Not sure	10

PN: ASK IF CODE 1 OR 2 SELECTED AT C9

C11. Please tell us why the COVID-19 pandemic has made it less likely that you will get the flu shot this coming fall (that is, between September and December of 2022)? (Select all that apply)

PN: RANDOMIZE

Because of social/physical distancing the seasonal flu almost disappeared last year	1
I don’t want to be exposed to COVID-19 in the process of getting the flu shot.	2
I worry about getting multiple vaccines	3
Because of social distancing I won’t be exposed to the seasonal flu	4
I Worry about potential side-effects	5
I Worry about how the flu shot could react with my COVID-19 vaccine	6
I Worry that it will make my COVID-19 vaccine less effective	7
Other (specify): ______	96
Don’t know/Not sure	99

PN: ASK IF CODE 1 SELECTED AT C2

C12. Did you encounter any of the following difficulties in scheduling an appointment for getting the flu shot last fall (that is, between September and December of 2021) due to the COVID-19 pandemic? (Select all that apply)

PN: RANDOMIZE

Limited appointment availability	1
Transportation to get the appointment was a problem	2
I didn’t know who to call to schedule an appointment	3
Concern about being exposed to COVID-19	4
No one could take care of my spouse/partner, children or other loved ones during the appointment	5
Lack of walk-in options	6
The vaccine was not offered at my usual/a convenient location	7
Other (specify): ________	96
I didn’t encounter any difficulties in scheduling an appointment **[MUTUALLY EXCLUSIVE]**	97
I did not take any action to get vaccinated last fall **[MUTUALLY EXCLUSIVE]**	98

Don’t know/Not sure **[MUTUALLY EXCLUSIVE]**	99
Preferred not to answer	99

C13. How worried, if at all, were you about getting the seasonal flu during the 2021-22 flu season (i.e., last flu season)?

Not at all worried<br>1	2	3	4	Very worried<br>5	Don’t know/<br>Not sure<br>98	Prefer not to say<br>99

C14. Were you more worried or less worried about getting the seasonal flu during the 2021-22 flu season (i.e., last flu season) compared to previous flu seasons?

A lot more worried about getting the flu during the 2021-22 flu season (i.e., last flu season)	1
A bit more worried about getting the flu during the 2021-22 flu season (i.e., last flu season)	2
No change	3
A bit less worried about getting the flu during the 2021-22 flu season (i.e., last flu season)	4
A lot less worried about getting the flu during the 2021-22 flu season (i.e., last flu season)	5
Prefer not to say	98

C15. Do you intend to receive the flu vaccine this coming fall (that is, between September and December of 2022)?

Yes, definitely	1
Yes, probably	2
Probably not	3
Definitely not	4
Don’t know/Not sure	98
Prefer not to say	99

C16. Moving forward, do you think you are more or less likely to get the flu vaccine over the next five years than you have in the past five years?

Much more likely	1
Somewhat more likely	2
No change	3

Somewhat less likely	4
Much less likely	5
Don't know/Not sure	98
Prefer not to say	99

PN: ASK IF CODE 1, 2, 4 OR 5 SELECTED AT C16

C17. Please briefly explain why you think you are **[PN: INSERT C16 RESPONSE]** to get the flu vaccine over the next five years than you have in the past five years

SECTION D: Flu vaccine Knowledge and Awareness

We would now like to understand your awareness and perceptions of different types of flu vaccines.

D1. Did you know that each year more than one type of flu vaccine is approved for use in Canada?

Yes	1
No	2

D2. Did you know that there are different influenza vaccines made for younger adults and for people 65 years of age and older?

Yes, definitely	1
Yes, vaguely	2
No	

PN: SHOW THE BELOW ON A SEPARATE SCREEN AFTER D2

There are, in fact, different influenza vaccines made for younger adults and for people 65 years of age and older. Enhanced influenza vaccines are designed to give older adults better protection against the flu.

D3. Did you know that there is more than one type of enhanced influenza vaccine made specifically for people 65 years of age and older?

Yes, definitely	1
Yes, vaguely	2
No	

PN: SHOW THE BELOW ON A SEPARATE SCREEN AFTER D3
There is, in fact, more than one type of enhanced influenza vaccine specifically formulated and approved for use in for people 65 years of age and older.

D4. Please indicate whether you agree with each of the following statements about enhanced influenza vaccines.

		Strongly disagree	Somewhat disagree	Somewhat agree	Strongly agree	Don't know/Not sure	Prefer not to say
1	Some enhanced vaccines are better than others	1	2	3	4	98	99
2	Enhanced vaccines help better protect older adults from the seasonal flu	1	2	3	4	98	99
3	It is important that older Canadians have access to enhanced vaccines	1	2	3	4	98	99
4	Enhanced vaccines should be available free of charge to any older Canadian who wants one	1	2	3	4	98	99

D5. If your province/territory offered enhanced influenza vaccines, would it make you more likely to get vaccinated against the flu?

Much more likely	1
Somewhat more likely	2
No change in likelihood	3
Somewhat less likely	4
Much less likely	5
Don't know/Not sure	98
Prefer not to say	99

D6. Please indicate whether you agree or disagree with each of the following statements about flu vaccination.
PN: RANDOMIZE

		Strongly disagree	Somewhat disagree	Somewhat agree	Strongly agree	Don't know/Not sure	Prefer not to say

1	The flu vaccine does not protect you against getting the flu	1	2	3	4	98	99
2	Sometimes, you can get the flu from the flu vaccine	1	2	3	4	98	99
3	The opinion of my family doctor, general practitioner, nurse practitioner and/or other health specialists is an important part of my decision when it comes to getting the flu vaccine	1	2	3	4	98	99
4	The flu vaccine is safe	1	2	3	4	98	99
5	I understand why the flu vaccine is recommended annually	1	2	3	4	98	99

D7. Please indicate whether you agree or disagree with each of the following statements.
PN: RANDOMIZE

		Strongly disagree	Somewhat disagree	Somewhat agree	Strongly agree	Don't know/Not sure	Prefer not to say
1	I trust the science behind COVID-19 vaccines	1	2	3	4	98	99
2	I trust the science behind vaccines	1	2	3	4	98	99
3	I trust the science behind flu vaccines	1	2	3	4	98	99
4	Vaccines are a lot more effective now than they used to be	1	2	3	4	98	99
5	I worry that COVID-19 vaccines are often rushed to market	1	2	3	4	98	99
6	I worry that flu vaccines are often rushed to market	1	2	3	4	98	99
7	I worry that getting a flu shot on top of a COVID-19 vaccine might overload my immune system	1	2	3	4	98	99

SECTION E: Demographics

E1. Please provide the first half of your postal code (e.g. K1K). [Open-end]

_____ (A2A)

E2. People living in Canada come from many different cultural and racial backgrounds. The following question will help us to better understand the experiences of the communities that we serve. Do you consider yourself to be . . . (Select all that apply)

First Nation	1
Inuit	2
Métis	3
Indigenous/Aboriginal (not included above)	4
Arab	5
Black (North American, Caribbean, African, etc.)	6
Chinese	7
Filipino	8
Japanese	9
Korean	10
Latin American	11
South Asian (East Indian, Pakistani, Sri Lankan etc.)	12
Southeast Asian (Vietnamese, Cambodian, Malaysian, Laotian, etc.)	13
West Asian (Iranian, Afghan, etc.)	14
White (North American, European, etc.)	15
Other (specify): _______________________	96
Prefer not to say	99

E3. Do you currently have a residential land-line telephone service at home?

Yes	1
No	2
Don’t know/Not sure	98
Prefer not to say	99

E4. What is your current marital status?

Single / never married	1
Married	2
Common law	3
Separated	4
Divorced	5
Widowed	6
Prefer not to say	99

E5. Including yourself, how many people live in your household, counting adults and children?

______ # people

- One person (myself)
- Prefer not to say

PN: ASK IF >1 AT E5

E6. How many people in each of the following age groups (if any) do you have living with you in your household?

<5 years old	___#
5-17 years old	
18-34 years old	___#
35-44 years old	___#
45-54 years old	___#
55-44 years old	___#
65 years of age or older	___#
Prefer not to say	99

SUM=E5

E7. What is the highest level of education you have completed?

Less than high school	1
High school	2
Some college or university	3
College graduate or CEGEP	4

Bachelor’s degree	5
Master’s or professional degree	6
Doctorate	7
Prefer not to say	99

E8. What is your current employment status?

Work for an employer full-time	1
Work for an employer part time	2
Self-employed	3
Unemployed	4
Student	5
Homemaker	6
Retired	7
Other (please specify) ______	96
Prefer not to say	99

E9. Which of the following best describes your current health insurance coverage?

Public / provincial coverage	1
Private insurance	2
No coverage	3
Don’t know/Not sure	98
Prefer not to say	99

E10. What is your annual household income (from all sources before taxes)?

Less than $20,000	1
$20,000-$39,999	2
$40,000-$69,999	3
$70,000-$99,999	4
$100,000-$119,999	5
$120,000 or more	6
Prefer not to say	99

E11. Which of the following location descriptions best defines where you live? (Select one only)

Rural (population of less than 50,000)	1

Small town (population between 50,000 – 250,000)	2
Large city (population from 250,000 – 1 million)	3
Metropolitan (population of 1 million or more)	4
Don't know/Not sure	5
Prefer not to say	6

E12. Were you born in Canada?

Yes	1
No	2
Prefer not to say	99

E13. On a scale of one to five, with one being poor and five being excellent, how would you rate your health?

One (poor)	1
Two (fair)	2
Three (good)	3
Four (very good)	4
Five (excellent)	5
Don't know/Not sure	6
Prefer not to say	99

PN: ASK IF NO AT E12
E14. In what year did you move to Canada?

Record year: XXXX
- Don't know/Not sure
- Prefer not to say

PN: ASK IF NO AT E12
E15. In which country were you born?

Afghanistan	1
Algeria	2
Bangladesh	3
Belgium	4
China	5
Colombia	6
France	7

Germany	8
Greece	9
Guyana	10
Hong Kong	11
India	12
Iran	13
Italy	14
Jamaica	15
Korea, Republic of (South Korea)	16
Lebanon	17
Netherlands	18
Pakistan	19
Philippines	20
Poland	21
Portugal	22
Romania	23
Russia	24
Sri Lanka	25
Taiwan	26
Trinidad and Tobago	27
Turkey	28
Ukraine	29
United Kingdom	30
United States	31
Vietnam	32
Other (please specify) ______	96
Don’t know/Not sure	98
Prefer not to say	99

Thank you for participating in the survey. This brings us to the end of the survey.
